# Phytomedicine Potential of *Oroxylum indicum* Root and Its Constituents: Targeting Alzheimer’s Disease

**DOI:** 10.3390/plants14020223

**Published:** 2025-01-15

**Authors:** Rattana Summat, Pornthip Waiwut, Supawadee Daodee, Natsajee Nualkaew, Khemjira Phemphunananchai, Puguh Novi Arsito, Yaowared Chulikhit, Orawan Montakantirat, Charinya Khamphukdee, Chantana Boonyarat

**Affiliations:** 1 Faculty of Pharmaceutical Sciences, Khon Kaen University, Khon Kaen 40002, Thailand; rattana_summat@kkumail.com (R.S.); csupawad@kku.ac.th (S.D.); nnatsa@kku.ac.th (N.N.); khemjira_ph@kkumail.com (K.P.); yaosum@kku.ac.th (Y.C.); oramon@kku.ac.th (O.M.); charkh@kku.ac.th (C.K.); 2 Faculty of Pharmaceutical Sciences, Ubon Ratchathani University, Ubon Ratchathani 34190, Thailand; pwaiwut79@yahoo.com; 3 School of Pharmacy, Faculty of Medicine and Health Sciences, Universitas Muhammadiyah Yogyakarta, Yogyakarta 55183, Indonesia; puguh.arsito@gmail.com

**Keywords:** baicalein, oroxylin A, chrysin, antioxidant, β-amyloid aggregation inhibition, anti-acetylcholinesterase, apoptosis

## Abstract

Alzheimer’s disease (AD) is a neurodegenerative condition characterized by a gradual decline in cognitive function, for which few effective treatments exist. This study investigated the neuroprotective potential of *Oroxylum indicum* root extract and its key constituents (baicalein, chrysin, oroxylin A) against AD hallmarks. The extract and its constituents exhibited antioxidant activity in the DPPH assay. They inhibited β-amyloid aggregation as measured by the thioflavin T assay and acetylcholinesterase activity using the Ellman method. In cell culture models, *O. indicum* extract showed an ability to protect neurons from the toxic effects of H_2_O_2_. Western blot analysis revealed the extract and its major active component, baicalein, downregulated pro-apoptotic markers (cleaved caspase-3, and BAX) upon H_2_O_2_ exposure. Furthermore, they reduced the expression of amyloidogenic proteins (BACE1) and phosphorylated tau. These findings suggest that *O. indicum* root extract, particularly baicalein, possesses multifaceted neuroprotective properties, targeting various aspects of AD pathogenesis, including oxidative stress, cholinergic dysfunction, β-amyloid formation, aggregation, and apoptosis. *O. indicum* root thus warrants further investigation as a promising source of therapeutic agents for AD.

## 1. Introduction

There have been several research studies on the potential of *Oroxylum indicum* for the prevention or treatment of several diseases. Various parts of *O. indicum* have been utilized for a long time for human treatment, particularly in Southeast Asian nations. The flowers and fruits of *O. indicum* were consumed as vegetables in Thai cuisine [1]. The root part has been reported to have antidiabetic, antiarthritic, constipating, diuretic, digestive, and expectorant properties [2]. The root bark of *O. indicum* was used to treat nasopharyngeal cancer, stomatitis, and tuberculosis [3]. In India, *O. indicum* root bark is one component in the Naga tribe formula, which was used to cure dysentery, diarrhea, jaundice, and rheumatism [4]. The root infusion of *O. indicum* is used to treat dysentery and diarrhea in Nepal [5]. Filipino folk medicine utilizes the root bark as a diaphoretic [6].

According to reports, flavonoids, alkaloids, tannins, glycosides, and quinones are the bioactive components discovered in the *O. indicum* plant. Flavonoids are the most abundantly detected in practically all parts of the plant [7,8,9,10]. Baicalein, oroxin A, oroxin B, chrysin, and oroxylin-A are flavonoids found in *O. indicum* [11]. These flavonoid compounds have been extensively described in many relationships to medicinal actions, especially anti-AD. There have been reports that the baicalein improved memory deficit in mice and rats [12,13]. Baicalin was found to have a protective effect against Aβ_1–42_-induced oxidative damage in neuronal and microglial cells [14,15]. Chrysin has been shown to protect neurons by exerting antioxidant, anti-apoptotic, and anti-inflammatory action [16]. Several prior studies revealed that oroxylin A is an antioxidant and anti-inflammatory substance [17,18]. Based on its component’s activities, *O. indicum* root extract might have the potential for use in Alzheimer’s disease (AD) therapy.

AD is the leading cause of dementia worldwide, accounting for 60–70% of cases [19,20]. According to estimates, there will be more than 50 million dementia sufferers globally with AD, accounting for 50 to 75 percent of those in 2020. There is a tendency for AD to rise considerably with age. AD affects 11.3 percent of people over the age of 65, and 34.6 percent of people over the age of 85 [21,22,23]. The patient has modest memory loss in the early stages of the disease, and as the disease advances, memory continues to deteriorate, accompanied by cognitive dysfunction and finally dementia [23,24]. The neuropathological hallmarks of AD are neurofibrillary tangles and amyloid plaques. Neurofibrillary tangles are formed by hyperphosphorylated tau protein, while amyloid plaques are composed of aggregated β-amyloid (Aβ) peptides [25]. In addition, mitochondrial malfunction, synaptic transmission failure, oxidative stress, and cell death all play a role in the development of AD [26,27,28,29]. There is presently no effective therapy available for preventing and/or curing AD [25].

However, there have been several efforts committed to identify the underlying processes of AD and uncover disease-modifying medicines that can stop AD progression [30]. Currently, the FDA has approved the medications rivastigmine, galantamine, donepezil, memantine, and Namzaric^®^ (a combined drug regimen of donepezil and memantine) for treating AD. Each of these medications has a different mechanism of action [31]. However, none of these medications have been proven to be effective in curing AD and they have several adverse effects following their use [32,33,34]. Hence, efforts have been made to find more effective medicines that can deliver more therapeutic efficacy while having fewer negative effects than current treatments. Researchers have looked for possible neuroprotective compounds in natural resources, as natural agents are typically safer than manufactured ones. Galantamine, for example, is a natural medicine derived from *Galanthus woronowii* that is now used to treat AD with other approved chemical treatments [35,36].

Therefore, the aims of this research are to investigate the effectiveness of the *O. indicum* root extract and its constituents to the following four pathological targets relevant to AD evolution, (i) cholinesterase function, (ii) antioxidant activity, (iii) Aβ aggregation, and (iv) neuroprotection in vitro. Furthermore, the level of proteins associated with neuronal cell death and AD pathogenesis is monitored in order to understand the mechanism of action.

## 2. Results

### 2.1. Total Flavonoid and Phenolic Contents Assessment

It has been well known that phenolic compounds and flavonoids derived from plants exhibit various pharmacological properties. Thus, the total phenolic and total flavonoid contents in the *O. indicum* root extract were determined. The results showed that the total phenolic and flavonoid contents of the *O. indicum* root extract were 306.51 ± 1.67 µg GAE/mg extract and 51.82 ± 5.31 µg QE/mg extract, respectively.

### 2.2. RP-HPLC Analysis of O. indicum Root Extract

**Figure 1:**
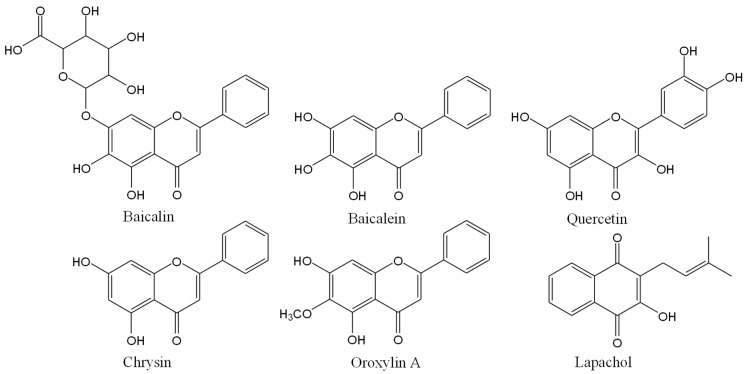
Chemical structure of standard markers used for HPLC analysis.

**Figure 2:**
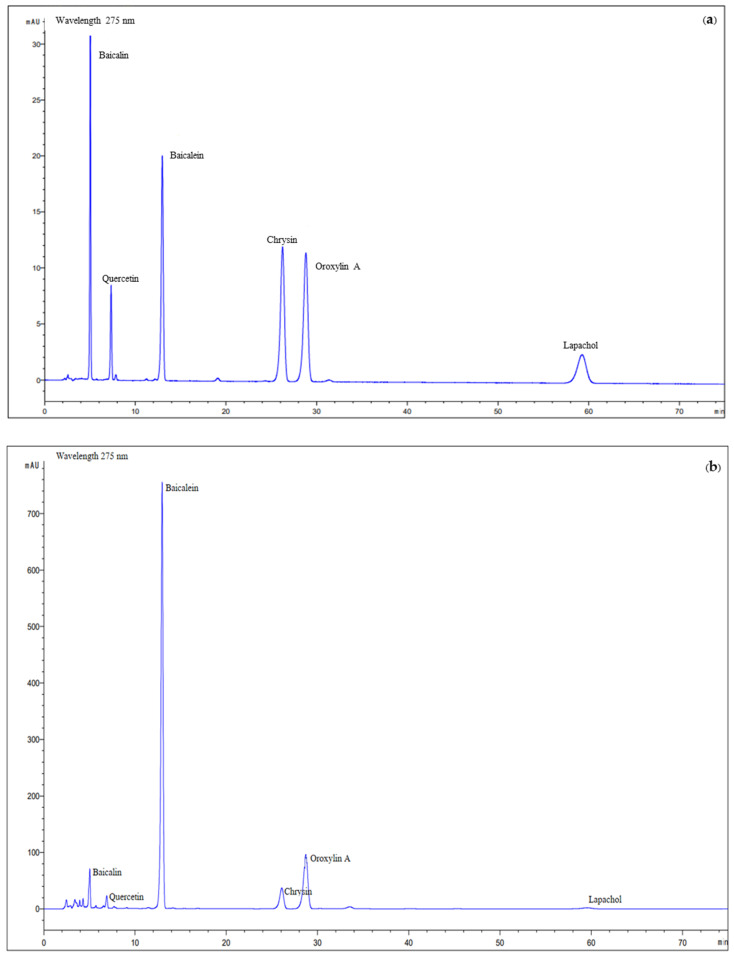
HPLC chromatograms of the reference standards (**a**) and the *O. indicum* root extract (**b**).

The six standard compounds used for HPLC analysis were baicalin, baicalein, quercetin, chrysin, oroxylin A, and lapachol (Figure 1). The HPLC chromatogram of the *O. indicum* root extract showed peaks of all major compounds that corresponded to the HPLC chromatogram of reference compounds. Baicalin, quercetin, baicalein, chrysin, oroxylin A, and lapachol provided peaks at a retention time of 5.04, 7.45, 12.98, 26.24, 28.81, and 59.26 min, respectively (Figure 2). The quantities of major compounds were calculated using a linear correlation equation for a range of 1.0–10.0 μg/mL (baicalin, baicalein, and chrysin), 0.5–10.0 μg/mL (oroxylin A and Lapachol), and 0.25–8.0 μg/mL (quercetin), with r^2^ values of more than 0.9996 for all six compounds, confirming the method’s linearity. The root extract of *O. indicum* contained the most baicalein (98.83 ± 0.49 mg/g), followed by oroxylin A, chrysin, baicalin, lapachol, and quercetin (58.10 ± 0.54, 18.01 ± 0.01, 15.28 ± 0.09, 6.24 ± 0.09, and 2.65 ± 0.20 mg/g, respectively).

### 2.3. Antioxidant Activities

One of the most important strategies used in the development of medications to treat AD is the decrease in oxidative stress. The antioxidant activity of ethanol extract of *O. indicum* root and its three major constituents including baicalein, chrysin and oroxylin A were examined using the DPPH radical scavenging method. The results are shown in Table 1. The results showed that the *O. indicum* root extract exhibited a potent DPPH radical scavenging capacity, with IC_50_ values of 29.00 ± 0.79 µg/mL. Trolox was utilized as a reference standard, having an IC_50_ value of 29.82 ± 1.59 µM for scavenging DPPH radicals. Baicalein demonstrated great radical scavenging action with an IC_50_ value of 19.08 ± 0.17 µM. Interestingly, it showed higher activity than the positive control, Trolox.

**Figure 3:**
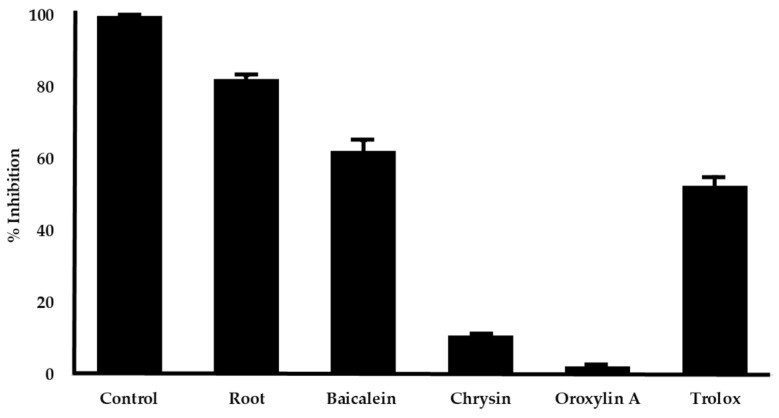
The DPPH radical scavenging effect of the *O. indicum* root extract and its major constituents. The *O. indicum* root extract at the concentration of 50 µg/mL and its constituents at equivalent amounts (baicalein 4.94 µg/mL, chrysin 0.90 µg/mL, and oroxylin A 2.90 µg/mL) were evaluated for radical scavenging activities by DPPH methods. The values obtained are reported as means ± SD (n = 3). Trolox at the concentration of 30 µM (7.5 µg/mL) was used as a positive control.

To investigate which component in root extract is mainly responsible for radical scavenging action, the *O. indicum* root extract at the concentration that inhibited 80% of radicals (50 µg/mL) and its major constituents at equivalent amounts, e.g., baicalein 4.94 µg/mL, chrysin 0.90 µg/mL, and oroxylin A 2.90 µg/mL, were evaluated for DPPH radical scavenging activity. Baicalein was the most potent radical scavenger, followed by chrysin and oroxylin A (Figure 3). Our findings suggest that baicalein is the primary contributor to the radical scavenging activity of *O. indicum* root extract.

### 2.4. Investigation of AChE Inhibitory Activity In Vitro

**Figure 4:**
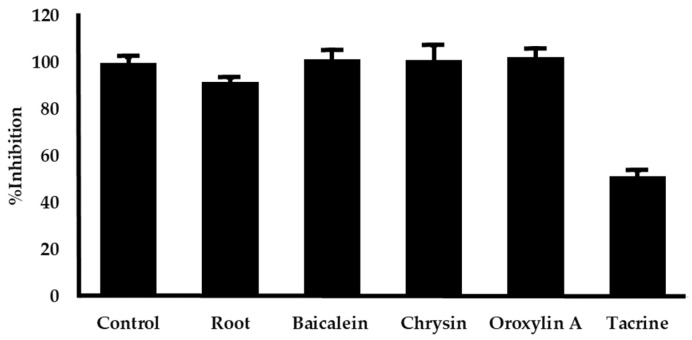
The effect of the *O. indicum* root extract and its major compounds (baicalein, chrysin, oroxylin A) on AChE activities. The *O. indicum* root extract of 3000 µg/mL and its constituents at equivalent amount found in the extract (baicalein 296.49 µg/mL, chrysin 54.03 µg/mL, and oroxylin A 174.30 µg/mL) were assessed for AChE inhibition using Ellman’s assay. Results are presented as means ± SD (n = 3). Tacrine at the concentration of 0.20 µM was used as a positive control.

The anti-acetylcholinesterase activity of *O. indicum* root extract and its major components were examined. The IC_50_ values measured are shown in Table 1. AChE function was suppressed by *O. indicum* root extracts with an IC_50_ value of 1702.88 ± 11.73 µg/mL. Tacrine served as a positive control since it has been shown to inhibit AChE with IC_50_ values of 0.20 ± 0.01 µM. Among the three constituents, chrysin exhibited the most potent inhibitory activity against AChE, followed by oroxyrin A and baicalein, as indicated by their respective IC_50_ values in Table 1. To further investigate the contribution of individual compounds to the extract’s activity, the inhibitory effects of the *O. indicum* root extract (3000 µg/mL) and its major components (baicalein 296.49 µg/mL, chrysin 54.03 µg/mL, and oroxylin A 174.30 µg/mL) at equivalent concentrations were assessed. As depicted in Figure 4, no significant differences in inhibitory activity were observed among the individual compounds at these concentrations. This suggests that the AChE inhibitory action of the *O. indicum* root extract may arise from the combined effects of all three major compounds.

### 2.5. In Vitro Investigation of Aβ Aggregation Inhibition

**Figure 5:**
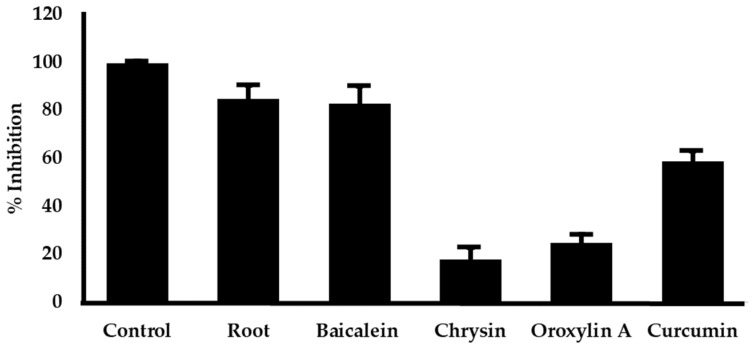
The effect of the *O. indicum* root extract and its major compounds (baicalein, chrysin, oroxylin A) on Aβ aggregation. The *O. indicum* root extract at 20 µg/mL and its major components at equivalent amounts found in the extract (baicalein 1.98 µg/mL, chrysin 0.36 µg/mL, and oroxylin A 1.16 µg/mL) were evaluated for their ability to inhibit Aβ_1–42_ aggregation using the ThT assay. Curcumin (10 µM, 3.68 µg/mL) served as a positive control. The control group contained no sample and no Aβ. The results are expressed as means ± SD (n = 3).

The *O. indicum* root extract and its major components were evaluated for Aβ aggregation inhibition. The results are presented with IC_50_ determinations (Table 1). The *O. indicum* root extracts inhibited Aβ aggregation with an IC_50_ value of 11.47 ± 0.46 µg/mL. Curcumin is used as a positive control, where it inhibits Aβ aggregation with an IC_50_ value of 4.97 ± 0.71 µM. Comparing among its constituents, baicalein showed the most potent with IC_50_ values of 2.27 ± 0.14 µM. Figure 5 represents the effect on Aβ aggregation of the extract and its major components at an equivalent amount. Our result exhibited that the inhibitory action of the extract might mainly come from baicalein.

### 2.6. Molecular Docking Study

**Figure 6:**
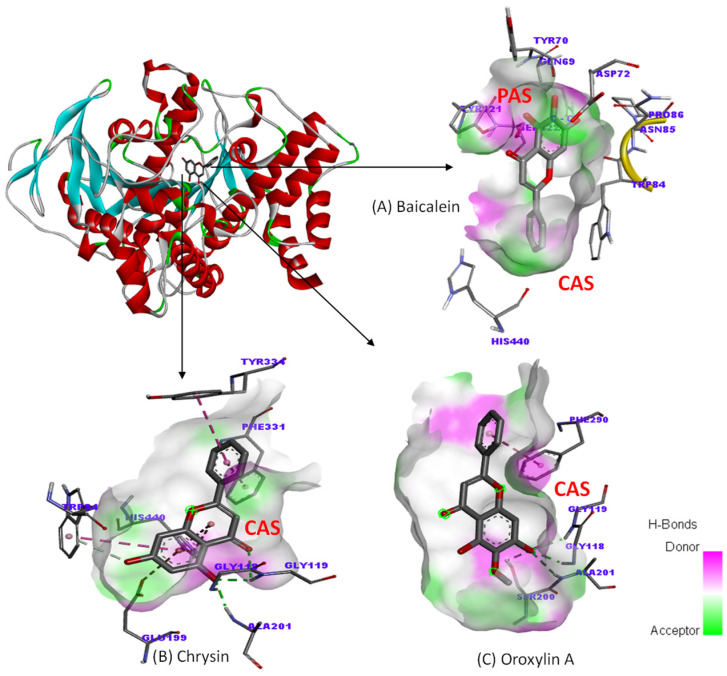
Binding mode of flavonoid derivatives from *Oroxylum indicum* root with AChE (**A**) baicalein, (**B**) chrysin, (**C**) oroxylin A.

**Figure 7:**
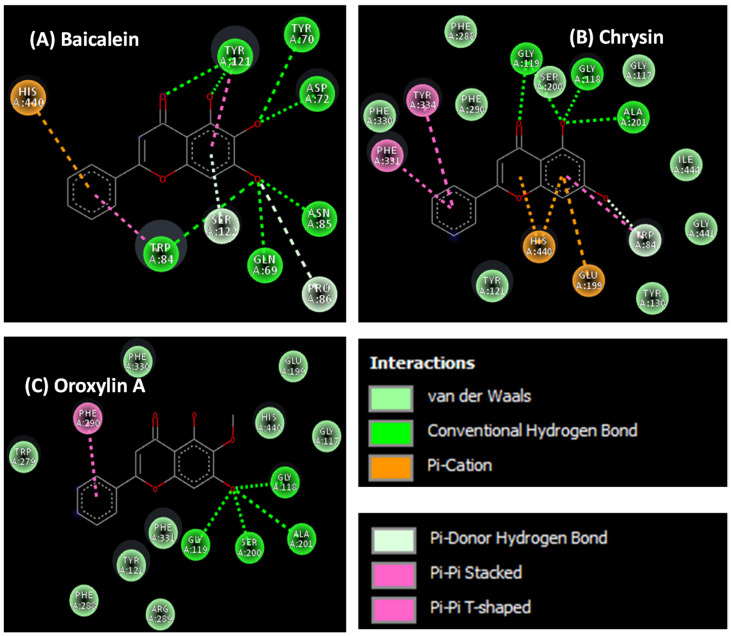
Binding interactions of flavonoid derivatives from *Oroxylum indicum* root with AChE (**A**) baicalein, (**B**) chrysin, (**C**) oroxylin A.

To understand the binding interaction between our ligands and their targets (AChE and Aβ), molecular docking was performed. This method can determine the position of the compound when it binds to the target. The binding orientation and the interaction profile of flavonoid compounds on the active site of AChE can be seen in Figure 6 and Figure 7. From the simulation results, chrysin and oroxylin A bind to a catalytic site (CAS); meanwhile, baicalein binds to a PAS.

**Figure 8:**
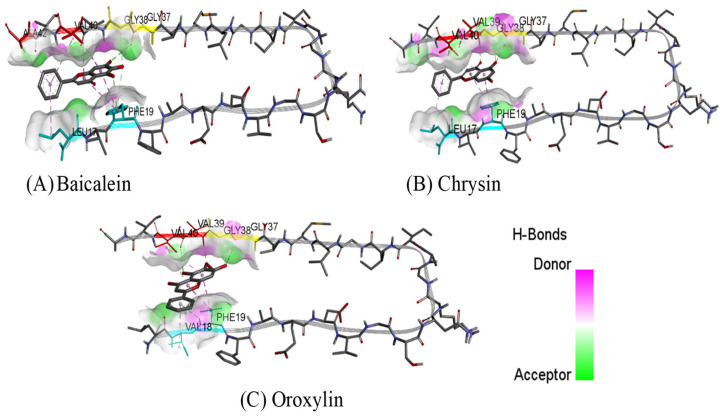
Binding interactions of flavonoid derivatives from *Oroxylum indicum* Root with Aβ_42_ (**A**) baicalein, (**B**) chrysin, (**C**) oroxylin A. The binding sites are coded with different colors (C terminal hydrophobic region: red; central hydrophobic region: cyan; residue contributes to two Aβ intermolecular binds: yellow).

**Figure 9:**
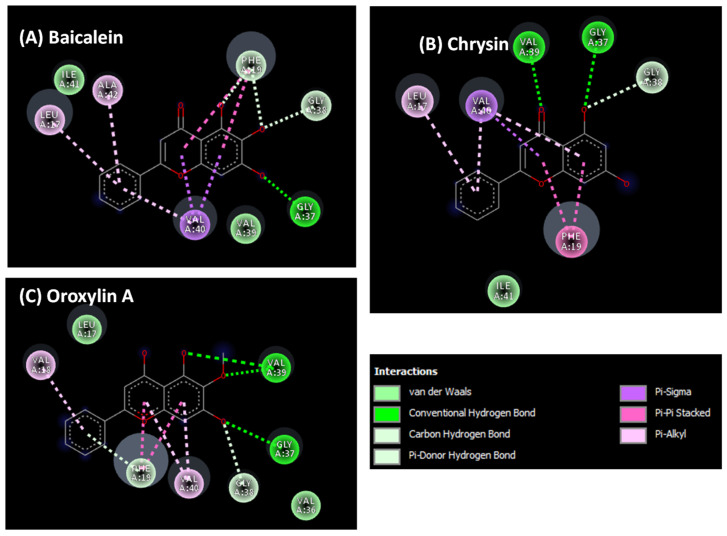
Binding interactions of flavonoid derivatives from *Oroxylum indicum* Root with Aβ_42_ (**A**) baicalein, (**B**) chrysin, (**C**) oroxylin A.

The binding mode and binding interaction profile of baicalein, chrysin, and oroxylin A to the Aβ can be seen in Figure 8 and Figure 9. The results show that all flavonoids can be bound to the C terminal hydrophobic residues, contributing to Aβ intermolecular binding.

### 2.7. Cytotoxicity

**Figure 10:**
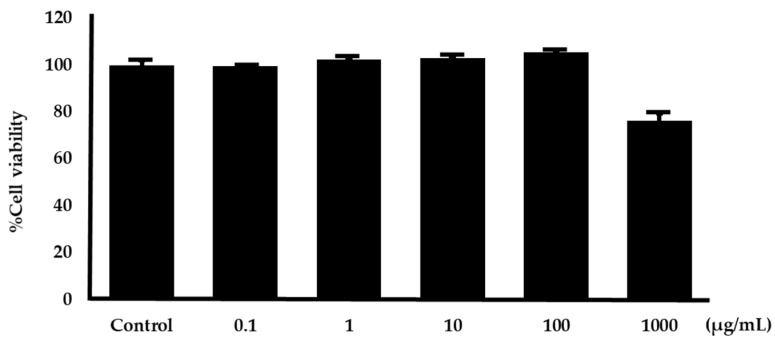
The cytotoxicity study results of *O. indicum* root extracts at various concentrations (0.1, 1, 10, 100, and 1000 µg/mL) to the SH-SY5Y cell line. The data were expressed as mean ± SD (n = 3).

An MTT assay was used to examine the cytotoxicity of the SH-SY5Y cell line impacted by *O. indicum* root extract. The neuronal cell was incubated with the *O. indicum* root extracts at concentrations of 0.1, 10, 100, and 1000 µg/mL for 2 h. The cell survival was measured by the MTT method, and the results demonstrated that *O. indicum* root extracts did not cause toxicity in the neuronal cell except at concentrations of 1000 µg/mL as shown in Figure 10.

### 2.8. Effect on Hydrogen Peroxide-Induced Cell Damage in Neuroblastoma Cells

**Figure 11:**
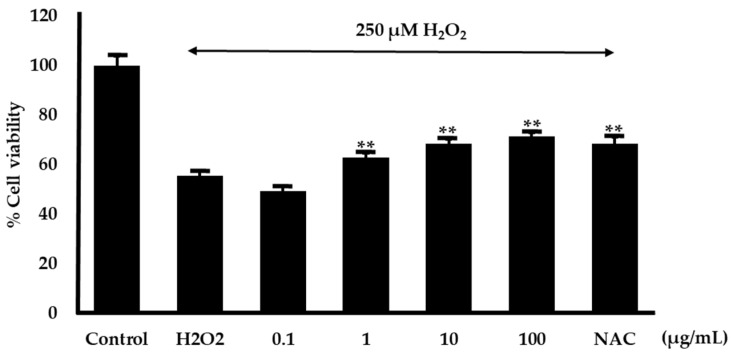
Protective effect of *O*. *indicum* root extracts against H_2_O_2_-induced oxidative stress in SH-SY5Y neurons. SH-SY5Y neurons were treated with 250 µM H_2_O_2_ in the presence or absence of *O. indicum* root extracts at various concentrations. N-acetyl cysteine (NAC) at 100 µg/mL was used as a positive control. Cell viability was assessed using an MTT assay. Data are presented as mean ± SD (n = 3). ** indicates a significant difference compared to the H_2_O_2_-only group (*p* < 0.01).

The neuroprotective effect of the *O. indicum* root extracts in mitigating oxidative stress was assessed using an MTT assay in SH-SY5Y neuroblastoma cells. Hydrogen peroxide (H_2_O_2_) was used to induce oxidative damage in neuron cells and N-acetyl cysteine (NAC) at 100 µg/mL was employed as a positive control. The findings revealed that the H_2_O_2_ concentration that induced 50% cell mortality was 250 µM. Thus, 250 µM H_2_O_2_ was chosen to induce oxidative damage in this experiment. *O. indicum* root extracts at concentrations of 0.1, 1.0, 10, and 100 µg/mL were treated in neuronal cells. The results found that the concentration of 1–100 µg/mL of *O. indicum* root extracts showed significantly increased cell viability (Figure 11).

### 2.9. Modulation of AD- and Apoptosis-Related Pathways in Neuroblastoma Cells

**Figure 12:**
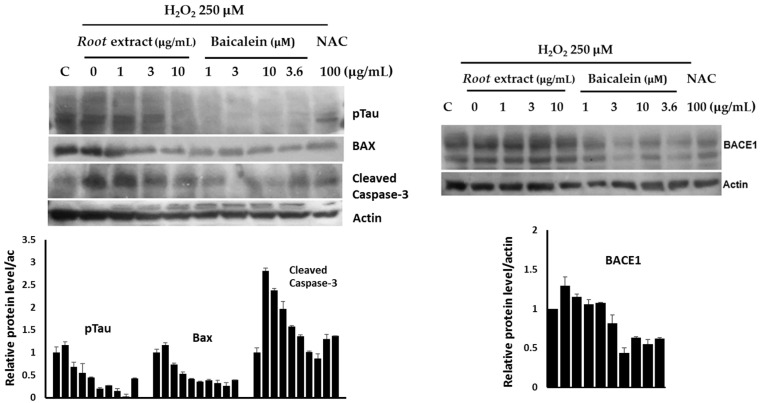
Levels of AD pathway-and neuronal cell death-related proteins in SH-SY5Y neuroblastoma cells exposed to 250 μM H_2_O_2_, with or without pretreatment with *O. indicum* root extract, baicalein and N-acetylcysteine (NAC) (a referent standard).

To investigate the neuroprotective effects of *O. indicum* root extract and its major active constituent, baicalein, we examined their impact on key proteins associated with neuronal cell death and AD pathways. We first assessed the ability of the extract and baicalein to protect neurons against oxidative stress-induced cell death. Pretreatment with both the extract and baicalein significantly downregulated the expression of pro-apoptotic proteins, Bax and cleaved caspase-3, in hydrogen peroxide-induced neuronal cells (Figure 12). These findings suggest that the extract and baicalein may protect neurons by inhibiting apoptotic pathways.

To explore the potential of *O. indicum* root extract and baicalein in mitigating AD-related pathologies, we examined their effects on key proteins involved in Aβ and tau protein pathways. We assessed the expression of β-site amyloid precursor protein cleaving enzyme 1 (BACE1), a key enzyme in Aβ generation. H_2_O_2_ treatment significantly upregulated BACE1 expression, while pretreatment with the root extract and baicalein effectively mitigated this increase. These findings suggest that the extract and its active constituent may inhibit Aβ formation. Neurofibrillary tangles (NFTs), composed of hyperphosphorylated tau protein, are another hallmark of AD. We observed that *O. indicum* root extract and baicalein significantly reduced the levels of phosphorylated tau protein. This indicates that the extract may exert its neuroprotective effects by modulating tau phosphorylation and aggregation. Overall, these findings demonstrate the neuroprotective potential of *O. indicum* root extract and baicalein in attenuating oxidative stress-induced neuronal cell death and AD-related pathologies.

## 3. Discussion

Due to the multifaceted nature of Alzheimer’s Disease, therapeutic agents that can target multiple pathological processes hold significant promise. Natural plants, with their diverse array of biological and chemical components, offer a potential source of multi-target drugs for this complex disease. *O. indicum*, with its reported constituents exhibiting various AD-related biological activities, emerges as a promising candidate.

The *O. indicum* root has been documented to contain a rich repertoire of phenolic and flavonoid compounds [7,8,9,10]. Previous studies have highlighted the anti-AD potential of phenolic compounds, which can inhibit the aggregation of Aβ peptides, a key pathological feature of AD [37]. Flavonoids, on the other hand, have demonstrated a range of biological activities relevant to AD, including anti-acetylcholinesterase, anti-Aβ aggregation, antioxidant, and anti-inflammatory properties [38]. In this study, we quantified the total phenolic and flavonoid contents of the *O. indicum* root extract and characterized its composition using six standard compounds including baicalin, baicalein, quercetin, chrysin, oroxylin A, and lapachol. HPLC analysis revealed that baicalein, oroxylin A, and chrysin are the predominant active ingredients in the extract.

To evaluate the anti-Alzheimer’s potential of the *O. indicum* root extract and its key compounds, we conducted a comprehensive assessment involving multiple biological assays relevant to AD pathology. These assays included antioxidant activity, Aβ aggregation inhibition, AChE inhibition, and neuroprotection against oxidative stress. To elucidate the mechanisms underlying these effects, we conducted molecular docking studies to investigate the binding interactions between the active constituents and their target proteins, AChE and Aβ. Additionally, we performed Western blotting analysis to evaluate the impact of the compounds on the expression of proteins related to neuronal death and AD pathology.

Oxidative stress, a condition resulting from an excess of free radicals that overwhelm the body’s antioxidant defenses, is a well-documented feature of brains affected by AD [39,40]. Antioxidant compounds can potentially mitigate the neurotoxic effects of oxidative stress and delay AD progression. Our results demonstrated that the *O. indicum* root extract and its constituents, baicalein and chrysin, possess significant antioxidant activity as measured by their ability to scavenge DPPH radicals. The presence of hydroxyl groups on the aromatic ring of these compounds appears to be crucial for their antioxidant activity, as previous studies have shown that these groups can donate electrons to free radicals, effectively neutralizing them [41]. Baicalein, with three hydroxyl groups, exhibited stronger antioxidant activity compared to chrysin, which has two hydroxyl groups. In contrast, oroxylin A, with a methoxy group neighboring the hydroxyl group, showed minimal DPPH radical scavenging activity up to 100 µg/mL, likely due to steric hindrance. When comparing the antioxidant activities of the *O. indicum* root extract and its individual constituents at equivalent concentrations, our results suggest that baicalein is the primary contributor to the extract’s antioxidant properties. This finding highlights the importance of baicalein as a potential therapeutic agent for AD, given its ability to mitigate oxidative stress.

The cholinergic pathway plays a critical role in various cognitive functions, and its dysfunction in AD is linked to cognitive impairment [42]. AChE inhibition is a primary therapeutic strategy for AD as it prevents the breakdown of acetylcholine (ACh), a neurotransmitter vital for learning and memory. This study demonstrated that the *O. Indicum* root extract exhibits AChE inhibitory activity with an IC_50_ value of 1.7 mg/mL. This is consistent with previous findings that flavonoids, including those found in the *O. Indicum* root extract, possess AChE inhibitory properties [43]. In vitro analysis revealed that baicalein, oroxylin A, and chrysin, the major components of the *O. Indicum* root extract, inhibit AChE activity. Chrysin was the most potent inhibitor, followed by oroxylin A and baicalein. Molecular docking studies elucidated the binding modes of these compounds to AChE. Chrysin bound to the catalytic site (CAS), directly interacting with the catalytic triad (Ser200, His440) and the anionic site (Trp84, Phe330), which is responsible for ACh hydrolysis. The methoxy group (-OCH3) at position 6 of oroxylin A hindered the binding of the hydroxy and carbonyl groups at positions 5 and 4, respectively, to the active site, resulting in decreased inhibitory activity compared to chrysin. Baicalein, however, bound to the peripheral site (PAS), blocking ACh access by interacting with key amino acids (Tyr70, Tyr121, Asp72). To further investigate the contribution of individual compounds to the extract’s activity, the inhibitory effects of the *O. indicum* root extract and its major components (baicalein, chrysin, and oroxylin A) were assessed at concentrations equivalent to their respective amounts in the extract. Our results indicated that no significant differences in inhibitory activity were observed among the individual compounds at these concentrations. This suggests that the AChE inhibitory action of the *O. indicum* root extract may arise from the combined effects of all three major compounds. This study highlights the potential of *O. Indicum* root extract as a source of potent AChE inhibitors. Baicalein, oroxylin A, and chrysin, the major components of the extract, contribute significantly to its anti-acetylcholinesterase activity. Understanding the molecular interactions of these compounds with AChE provides valuable insights for the development of novel AD therapeutics.

Aβ plaque deposition in the brain is a key hallmark of AD pathogenesis [25]. Consequently, preventing or decreasing the generation of Aβ plaques has been a primary focus of numerous therapeutic approaches currently being developed or tested in clinical trials. Senile plaques are primarily composed of Aβ_1–40_ and Aβ_1–42_, with Aβ_1–42_ showing greater toxicity due to its increased propensity for self-assembly into fibrils [44,45]. Accordingly, interfering with Aβ_1–42_ aggregation may be a promising therapeutic approach for AD treatment. This study investigated the ability of *O. indicum* root extract and its key flavonoids (baicalein, oroxylin A, and chrysin) to inhibit Aβ_1–42_ aggregation. The ThT fluorescence assay revealed that *O. indicum* extract effectively inhibited Aβ_1–42_ aggregation. Interestingly, all three individual flavonoids displayed comparable activity to curcumin (a known anti-amyloidogenic compound) at micromolar concentrations. To identify the primary compound responsible for this inhibition, we evaluated the effect of the root extract at a concentration that inhibited 80–90% of Aβ aggregation and its major components at equivalent amounts. Our results suggest that baicalein is the primary contributor to the anti-amyloidogenic activity of *O. indicum* root extract. Molecular docking studies further elucidated the potential binding sites of these flavonoids. Docking results identified the C terminal hydrophobic segment (residues 37–40) of Aβ_1–42_ as a common target for all three flavonoids. This region is crucial for initiating fibril nucleation and formation [46]. Notably, the flavonoids formed hydrogen bonds with Gly37 and Gly38, residues implicated in early intermolecular interactions [47]. Additionally, π–π stacking interactions with Phe19, a residue critical for β-sheet stabilization, were observed. Baicalein exhibited stronger inhibitory activity than chrysin and oroxylin A. This might be attributed to its additional interaction with Ile41 and Ile42, amino acids crucial for paranuclei formation and oligomer assembly, respectively [47,48]. Our findings demonstrate that *O. indicum* root extract and its constituent flavonoids, baicalein, oroxylin A, and chrysin, possess promising potential as inhibitors of Aβ aggregation. These compounds effectively target critical regions within the Aβ_1–42_ peptide, disrupting its aggregation process and potentially mitigating the progression of AD.

Reactive oxygen species (ROS) play a pivotal role in neuronal cell death, particularly through apoptosis. In this study, we demonstrated that pre-treatment of SH-SY5Y cells with the *O. indicum* root extract significantly enhanced their survival against H_2_O_2_-induced toxicity. This neuroprotective effect is likely associated with the antioxidant properties of the extract, which can neutralize harmful ROS and prevent oxidative damage. However, other mechanisms may also contribute to the observed protection. To elucidate the underlying mechanisms of *O. indicum* root extract-mediated neuroprotection, we examined the protein levels of apoptotic markers using Western blotting. Our results revealed that pretreatment with the extract and its major active component, baicalein, led to a downregulation of pro-apoptotic proteins, including Bax, and cleaved caspase-3. Previous research has implicated the mitochondrial apoptotic pathway as a key mechanism in oxidative stress-induced neuronal death in H_2_O_2_-treated SH-SY5Y cells [49]. Excessive ROS production can disrupt mitochondrial membrane integrity, leading to the release of cytochrome C, which in turn activates apoptotic proteins like caspase-3 and ultimately triggers cell death. Our findings align with the previous report, as we observed increased cleavage of caspase-3 and upregulation of Bax in H_2_O_2_-treated cells. Notably, the *O. indicum* root extract and baicalein protected neuronal cells by preventing these apoptotic events, as evidenced by the downregulation of cleaved caspase-3 and Bax. Our results suggest that the *O. indicum* root extract and baicalein exert neuroprotective effects via both antioxidant and anti-apoptotic mechanisms.

The accumulation of Aβ plaques and neurofibrillary tangles (NFTs) are hallmarks of AD. Oxidative stress has been implicated in the pathogenesis of AD, as it can influence the metabolism of amyloid precursor protein (APP) and tau protein [50]. Our results demonstrate that oxidative stress, induced by H_2_O_2_, significantly upregulated the expression of BACE1, a key enzyme involved in the amyloidogenic processing of APP [51]. This upregulation is indicative of increased Aβ production, which is a major contributor to AD pathogenesis. However, pretreatment with *O. indicum* root extract and baicalein effectively attenuated the H_2_O_2_-induced increase in BACE1 expression. These findings suggest that *O. indicum* root extract and baicalein may exert neuroprotective effects by inhibiting Aβ production. In addition to Aβ plaques, NFTs, composed of hyperphosphorylated tau protein, are another hallmark of AD. Tau protein plays a crucial role in maintaining microtubule stability [52]. However, the aberrant phosphorylation of tau leads to its aggregation into NFTs, which disrupt neuronal function and contribute to neurodegeneration. Our study revealed that *O. indicum* root extract and baicalein significantly reduced the levels of phosphorylated tau protein. This finding suggests that the extract may exert its neuroprotective effects by modulating pathways involved in tau phosphorylation and aggregation. Our study provides evidence that *O. indicum* root extract and its active constituent, baicalein, may have therapeutic potential for AD.

The overall results demonstrate that *O. indicum* root extract and its major components, especially baicalein, exert multifaceted effects on key pathways implicated in AD, including mitigating oxidative stress, inhibiting Aβ formation, reducing tau protein hyperphosphorylation, and modulating AChE activity. This multi-target approach holds promise for addressing the complex and multifaceted nature of AD pathology. These results position *O. indicum* extract and baicalein as promising candidates for further investigation as therapeutic agents for AD. However, in vivo studies are warranted to validate their therapeutic efficacy of the extract in the context of AD treatment.

## 4. Materials and Methods

### 4.1. Chemicals and Reagents

The root of *O. indicum* was obtained from Roi-et province, Thailand, and identified by Dr. Prathan Luecha. The voucher specimen (CY 4303) is deposited in the Herbarium of Khon Kaen University’s Faculty of Pharmaceutical Science. The standard chemicals baicalein, baicalin, chrysin, and oroxylin A were acquired from Biopurify, Bangkok, Thailand, and lapachol was purchased from Sigma-Aldrich (SM Chemical supplies Co., Ltd., Bangkok, Thailand). HPLC grades of methanol and acetonitrile were purchased from Merck (KSK Chemicals and Laboratory Appliance, Khon Kaen, Thailand).

### 4.2. Preparation of O. indicum Root Extracts

The crude extract of *O. indicum* root was prepared by weighing 50 g of dried *O. indicum* root powder, soaking it well in 400 mL of ethanol (1:8 *w*/*v* ratios), and macerating it for seven days. Then, the filtrate was concentrated by a rotary evaporator and dried by lyophilizer to yield a 3.44 percent dry weight of *O. indicum* root extract.

### 4.3. Analysis of Total Phenolic and Total Flavonoid Levels

The total phenolic level of the *O. indicum* root extract was analyzed using a modified method from [53]. Briefly, the extract was dissolved in ethanol, and the solution was supplemented with 75 µL of Folin–Ciocalteu reagent. After a 5 min incubation, 75 µL of 7.5% sodium carbonate solution was added. Following a 2 h incubation, the absorbance at 700 nm was recorded. The total phenolic content was determined using a calibration curve constructed with gallic acid and expressed as micrograms of gallic acid equivalents per milligram of crude extract (µg GAE/mg CE).

The method for the determination of total flavonoid content was adapted from [53]. A total of 20 microliters of the *O. indicum* root extract at the concentration of 1 mg/mL was combined with 20 µL of CH_3_COONa (100 g/L), 15 µL of AlCl_3_ solution (2.5%), and 145 µL of water in a 96-well plate. After a 15 min incubation, the absorbance at 450 nm was measured. The content of total flavonoid was represented as micrograms of quercetin equivalents per milligram of crude extract (µg QE/mg CE) based on a calibration curve prepared with quercetin.

### 4.4. RP-HPLC Determination of the Major Compound Quantities in O. indicum Root Extracts

A reversed-phase HPLC technique adapted from [54] was applied to assess the chemical component composition of the root portion extracts. For measurement of baicalin, quercetin, baicalein, chrysin, oroxylin A, and lapachol, an Agilent Technologies HPLC system with a UV detector (Santa Clara, CA, USA) was used with a Platisil ODS column (4 × 250 mm, 5 µm). Water, methanol, acetonitrile, and orthophosphoric acid (60:30:38:1, *v*/*v*/*v*/*v*) were utilized as a ternary isocratic solvent solution. The detection wavelength was set to 275 nm and the flow rate was maintained at 1.0 mL/min. The injection volume was set at 10 µL. The HPLC validation of the major compounds in *O. indicum* root will be reported elsewhere. The quantities of major compounds (baicalin, baicalein, chrysin, oroxylin A, lapachol, and quercetin) were determined using a linear regression analysis within the following concentration ranges: 1.0–10.0 μg/mL (baicalin, baicalein, and chrysin), 0.5–10.0 μg/mL (oroxylin A and Lapachol), and 0.25–8.0 μg/mL (quercetin). All calibration curves exhibited excellent linearity (r^2^ > 0.9996). By comparing the calibration curves of the reference standards utilized, the amounts of baicalin, quercetin, baicalein, chrysin, oroxylin A, and lapachol in the root portion extracts were estimated.

### 4.5. In Vitro Antioxidant Activity Investigation

The antioxidant activities of the *O. indicum* root extract and its chemical constituents (baicalein, chrysin, oroxylin A) were investigated using the 1,1-diphenyl-2-picrylhydrazyl (DPPH) method, adapted from [55]. A total of 50 microliters of various concentrations of the crude extract or individual compounds dissolved in ethanol were combined with 50 microliters of DPPH solution (0.2 mM). After 30 min incubation period, the absorbance at 520 nm was measured using a microplate reader. The DPPH radical scavenging ability was calculated according to the following equation:

Percentage inhibition = [(A_DPPH_ − A_sample_)/(A_DPPH_ − A_control_)] × 100

where A_DPPH_, A_sample_, and A_control_ are the absorbance values of DPPH solution, sample, and control, respectively. Crude extract’s antioxidation activities would be reported at the 50% inhibitory concentration (IC_50_) of free radical scavenging.

### 4.6. In Vitro Assessment of Acetylcholinesterase Inhibition

The anti-acetylcholinesterase activity of the extract of *O. indicum* root and its three constituent compounds was evaluated using a modified Ellman’s method [56]. A microplate-based assay was conducted in triplicate, comprising (i) 25 µL of 1 mM acetylthiocholine iodide, (ii) 50 µL of 0.1 M phosphate buffer (PBS), (iii) 25 µL of the sample, and (iv) 125 µL of 1 mM dithiobisnitrobenzoic acid. Electric eel-type VI-S AChE (0.2 Units/mL) was added. The control group lacked both the sample and enzyme. The absorbance at 405 nm was monitored continuously for 5 min, with readings taken every 30 s [57]. Tacrine served as a reference standard. Enzymatic activity and percent inhibition were assessed. The following equation was used to determine inhibitory activity:

Percentage inhibition = [(A_E_ − As)/(A_E_ − A_C_)] × 100

where A_E_, A_C,_ and As represent the enzymatic activities of the enzyme, control, and sample, respectively. The AChE inhibitory activity of the samples was expressed as the half-maximal inhibitory concentration (IC_50_).

### 4.7. In Vitro Evaluation of Aβ Aggregation Inhibition

The aggregation of Aβ_1–42_ was monitored using a thioflavin-T (ThT) fluorescence assay [58]. Ten micromolars of curcumin served as a positive control. In 96-well plates, 20 µL of 10 μM Aβ_1–42_ in 10 mM PBS was incubated with 5 µL of the *O. indicum* root extract, its constituent compounds (baicalein, chrysin, oroxylin A) at various concentrations, or curcumin at 10 μM at 37 °C. Following a 48 h incubation period, the mixtures were treated with 175 µL of 5 µM ThT solution prepared in a glycine/NaOH buffer at pH 8.5. Fluorescence measurements were taken at an excitation/emission wavelength of 446 nm and 490 nm. The percentage inhibition of aggregation was calculated as follows:

Percentage inhibition of aggregation = (1 − IFsample/IFcontrol) × 100%

where IFsample and IFcontrol represent the fluorescence intensities of the sample and control, respectively.

### 4.8. Molecular Docking

The β-amyloid protein and acetylcholine esterase were built using the X-ray crystal PDB with codes 2BEG and 1EVE, respectively [59,60]. Molecular docking studies were carried out by utilizing Autodock 4.2.6 program [61]. Autodock Tools version 1.5.6 was used for assigning the charges to the targets and ligands, identifying the torsion of the ligands, and adding the solvation parameters. The grids were generated by AutoGrid 4 with a grid space of 0.375 Å. The grid box dimensions for AChE and Aβ_1–42_ fibrils were established with sizes of 80 × 70 × 70 Å and 120 × 60 × 40 Å, respectively [58]. A population size of 100 individuals and 100 ligand orientation runs was employed. The maximum number of allowed assessments was set to 27,000, with a maximum of 1,000,000 energy evaluations. The best conformation was determined to be the one with the lowest docked energy. Interactions within the docking complex were analyzed using BIOVIA Discovery Studio 2017 [62,63]. 

### 4.9. Cellular Toxicity Assessment

Human neuroblastoma SH-SY5Y cells were maintained in a humidified incubator with 5% carbon dioxide. The cells were cultured in a 1:1 mixture of F12 and DMEM supplemented with 10% FBS. To assess cytotoxicity, *O. indicum* root extracts at concentrations of 0.1–1000 µg/mL were incubated with SH-SY5Y cells for 2 h in 96-well plates. Subsequently, methylthiazolyldiphenyl-tetrazolium bromide, MTT (0.5 mg/mL) was added, followed by a 2 h incubation period at 37 °C. The resulting formazan crystals, produced by viable cell enzymes, were dissolved in 100 µL of 100% dimethyl sulfoxide (DMSO). The absorbance at 570 nm was recorded and the percentage of cell survival was calculated as follows [64]:

% Cell survival = (OD_Sample_/OD_blank_) × 100

where OD_Sample_ and OD_blank_ are the absorbances at 570 nm of sample and blank, respectively.

### 4.10. Neuroprotective Effect Against Hydrogen Peroxide

For neuroprotective studies, SH-SY5Y cells were seeded onto 96-well plates with a density of 2.5 × 10^5^ cells/mL. After 48 h, the cells were pretreated with *O. indicum* root extracts at concentrations ranging from 0.1 to 100 µg/mL for 2 h, followed by 2 h incubation with hydrogen peroxide (H_2_O_2_ Cell survival was assessed using the MTT assay, following the same protocol as the cytotoxicity test [65]). The percentage of cell survival was calculated as follows:

% Cell survival = (OD_Sample_/OD_blank_) × 100

where OD_Sample_ and OD_blank_ are absorbance values of the sample and blank at 570 nm, respectively.

### 4.11. Modulation of AD- and Apoptosis-Related Pathways in Neuroblastoma Cells

To investigate the neuroprotective effects of *O. indicum* root extract and its major active constituent, baicalein, we examined their impact on key proteins involved in neuronal cell death and AD pathways. Neuronal cells were pretreated with the extract (1, 3, and 10 µg/mL) or baicalein (1, 3, and 10 µM) for 30 min, followed by induction of oxidative stress with hydrogen peroxide (H_2_O_2_). Baicalein at a concentration of 3.6 µM, which is equivalent to the amount found in the 10 µg/mL root extract, was also included in the study. NAC (100 µg/mL) was used for the standard substance. For the experiment, SH-SY5Y cells (1 × 10^5^ cells/well) were seeded onto a 6-well plate and cultured for 2 days. Subsequently, cells were treated with various concentrations of root extracts or baicalein, the most active component, for 2 h. After washing unabsorbed chemicals, the cells were exposed to 250 μM H_2_O_2_ for 5 min. Treated cells were lysed with an ice-cold lysis solution. The supernatant was obtained after centrifuging the lysate at 13,500 rpm for 10 min at 4 °C. The Bradford test was used to determine the total protein content. Proteins were separated on a polyvinylidene difluoride membrane using SDS-PAGE. After blocking with BlockAce, the membrane was probed with primary antibodies against BAX, cleaved caspase-3, pTau, and BACE1. Secondary antibodies coupled with horseradish peroxidase were used for detection, followed by enhanced chemiluminescence [66].

### 4.12. Statistical Analysis

All in vitro experiments were conducted in triplicate, and the results are presented as mean ± standard deviation (SD). Statistical analysis was performed using one-way analysis of variance (ANOVA) followed by Tukey’s post hoc test. A *p*-value of <0.05 was considered statistically significant for all analyses.

## 5. Conclusions

This study provides compelling evidence for the neuroprotective potential of *Oroxylum indicum* root extract and its bioactive compound, baicalein, against Alzheimer’s disease hallmarks. Our findings demonstrate that both the extract and baicalein possess potent antioxidant activity, inhibit Aβ aggregation and acetylcholinesterase activity, and safeguard neurons against oxidative stress. Moreover, they modulate the expression of key proteins implicated in amyloidogenesis and tau phosphorylation, suggesting a multi-target approach to AD pathogenesis. Importantly, the extract and baicalein promoted neuronal survival by downregulating pro-apoptotic markers. These results position *O. indicum* extract and baicalein as promising candidates for further development as therapeutic agents for AD. In vivo studies are warranted to validate the therapeutic efficacy of the extract in the context of AD treatment.

## Figures and Tables

**Table 1:** The in vitro antioxidant, anti-AChE, and anti-Aβ aggregation investigation of the *O. indicum* root extract and its constituents. Data are represented as mean ± SD (n = 3). Different letters within the same column indicate statistically significant differences at *p* < 0.05.

Samples	IC_50_ (µM)		
	DPPH	AChE	Aβ Aggregation
Root extract (µg/mL)	29.00 ± 0.79	1702.88 ± 11.73	11.47 ± 0.46
Baicalein	19.08 ± 0.17 ^a^	159 ± 1.15 ^d^	2.27 ± 0.14 ^a^
Chrysin	63.28 ± 0.89 ^c^	45.87 ± 1.32 ^b^	5.86 ± 0.16 ^b^
Oroxylin A	>100	86.94 ± 1.85 ^c^	8.19 ± 0.28 ^c^
Trolox	29.82 ± 1.59 ^b^	-	-
Tacrine	-	0.20 ± 0.01 ^a^	-
Curcumin	-	-	4.97 ± 0.71 ^b^

## Data Availability

The original contributions presented in this study are included in the article. Further inquiries can be directed to the corresponding author.
